# Healthcare costs of paternal depression in the postnatal period

**DOI:** 10.1016/j.jad.2011.04.005

**Published:** 2011-09

**Authors:** Ijeoma P. Edoka, Stavros Petrou, Paul G. Ramchandani

**Affiliations:** a Centre for Health Economics, Alcuin block A, University of York, York YO10 5DD, United Kingdom; b Warwick Clinical Trials unit, University of Warwick, Conventry, CV4 7AL, United Kingdom; c University of Oxford, Department of Psychiatry, Warneford Hospital, Headington, Oxford OX3 7JX, United Kingdom

**Keywords:** Healthcare costs, Paternal postnatal depression, National Health Service (NHS), Community care service costs

## Abstract

**Background:**

There is growing evidence that fathers experience depressive symptoms following the birth of a child. The aim of this study was to estimate the healthcare costs of paternal postnatal depression, thereby informing research into cost-effective preventative and treatment interventions for the condition.

**Methods:**

Data on healthcare resource-use over the first 12 months postpartum was collected from 192 fathers recruited from two postnatal wards in southern England. Three groups of fathers were identified: fathers with depression (n = 31), fathers at high risk of developing depression (n = 67) and fathers without depression (n = 94).

**Results:**

Mean father–child dyad costs were estimated at £1103.51, £1075.06 and £945.03 (£ sterling, 2008 prices) in these three groups, respectively (P = 0.796). After controlling for potentially confounding factors, paternal depression was associated with significantly higher community care costs.

**Conclusion:**

This study provides useful preliminary insights into the healthcare costs associated with paternal depression during the postnatal period.

**Limitation:**

The small sample size may, in part, account for the failure to detect statistically significant differences in mean costs between study groups for most cost categories.

## 1 Introduction

The focus of much research on postnatal depression has been on mothers. However, there is growing evidence that fathers also experience depressive symptoms following the birth of a child (Paulson and Bazemore, 2010; Schumacher et al., 2008). In addition, paternal depression in the postnatal period is associated with an increased risk of adverse behavioural and emotional outcomes and psychiatric problems in the child (Ramchandani et al., 2005, 2008).

Although it has been demonstrated that postnatal depression in mothers is associated with increased utilisation of health and social care services, resulting in higher economic costs (Petrou et al., 2002a), it is not clear whether depression in fathers during the postnatal period is also associated with increased economic costs. The aim of this study was to estimate, for the first time, the cost of paternal depression in the postnatal period, using self-reported resource-use data collected alongside a longitudinal study. Costs were estimated from the health system perspective as recommended by the National Institute for Health and Clinical Excellence (NICE, 2008).

## 2 Method

Fathers were recruited between the years 2006 and 2008 in the postnatal wards of the John Radcliffe Hospital, Oxford and the Milton Keynes General Hospital. At 7 weeks postpartum, a screening questionnaire for depression, the Edinburgh Postnatal Depression Scale (EPDS) (Matthey et al., 2001), was sent to fathers for completion. A detailed description of the study recruitment methods, assessments and ethical procedures can be found elsewhere (Edmondson et al., 2010).

At 3 months postpartum, one in four fathers screening negative (EPDS score < 10) were randomly selected for further assessment using a random numbers table. The random sample of fathers screening negative and all fathers screening positive (EPDS ≥ 10) were further assessed for Major Depressive Disorder using the Structured Clinical Interview for Diagnostic and Statistical Manual of Mental Disorders (DSM)-IV (SCID) (Sanchez-Villegas et al., 2008). This structured interview was then repeated at 12 months postpartum. Three groups of fathers were established: those with depression during the postnatal period (group 1), those who were not currently depressed but who had been at risk of developing depression because they had a previous history of depression or scored highly (≥ 10) on the EPDS (group 2) and those without depression (group 3). The three groups of fathers did not differ significantly in terms socio-demographic characteristics. The average age of fathers in groups 1, 2 and 3 was approximately 36, 35 and 35 years, respectively. The three groups of fathers were mainly of white ethnic or European origin (approximately 97%, 94% and 89%, respectively). High proportions of fathers in the three groups (97%, 100% and 97%, respectively) were either married or living together. Approximately 90%, 93% and 94% of fathers in groups 1, 2 and 3, respectively, were in full time employment. Approximately 58%, 66% and 57% of fathers in groups 1, 2 and 3, respectively, had either a degree or a postgraduate degree, whereas approximately 16%, 18% and 25% of fathers in the three groups had either A-levels, GCSEs or equivalents or no qualifications.

Data on health service resource-use was identified and collected as an integral component of the longitudinal study (Edmondson et al., 2010). At 12 months postpartum, fathers were asked to complete a questionnaire consisting of twenty four close-ended questions pertaining to their use of various healthcare services since the birth of their child. The main categories of services utilised included community care services, day care hospital services and hospital out- and in-patient services. For community care services, such as general practitioner (GP) contacts, fathers were asked to identify the location (home or care centre) in which the care was provided, the total number of contacts and the average duration per contact. For inpatient hospital services, fathers were asked to give the total number of days of each admission, by type of inpatient ward. For day care hospital and outpatient hospital services, fathers were asked to give the total number of separate attendances. Furthermore, a set of nine close-ended questions were completed by fathers on paediatric/child care services utilised by their respective children during the first 12 months of the child's life.

The cost of each item of resource-use was calculated as a product of the quantity of resource-use and its corresponding unit cost. Unit costs were obtained from two principal sources, the Unit Costs of Health and Social Care, published by the University of Kent's Personal and Social Services Research Unit (Curtis, 2008), and the English Department of Health NHS 2008 reference costs (NHS Reference Costs, 2008). All items of resource-use were valued at 2008 prices and expressed in pounds sterling (£). Costs incurred by fathers as well as costs incurred by their respective children were combined to obtain total father–child dyad costs. Approximately 17% of the data on resource-use items were missing. This was due to the failure of the participants to complete all relevant aspects of the study questionnaire. In order to avoid biassed estimation of the mean cost differences, the multiple imputation method was used to deal with missing cost values (Briggs et al., 2003). All individual cost variables (GP costs, psychologist costs, etc.), as well as relevant socio-demographic variables and EPDS scores, were used in the multiple imputation process. Fathers with missing data did not differ significantly from those with complete data in terms of socio-demographic status (age, marital status, academic qualification, employment status and ethnicity) and EPDS score.

Differences in mean costs between the three study groups were compared using a one-way ANOVA test. Mean cost differences between fathers with and without depression were assessed using a Student's t-test. The 95% confidence intervals surrounding mean cost differences between these two study groups were estimated using the non-parametric bootstrap method based on 2000 bias-corrected bootstrap replications (Chernick, 2008). The association between the fathers' depressive status and healthcare costs was further assessed using generalised linear models (GLMs) (Barber and Thompson, 2004). Total father–child dyad costs, costs of community care and the costs of day care hospital services acted as dependent variables in three separate GLMs. The main independent variable was a categorical variable of paternal postnatal depression status (categorised as depressed, high risk of developing depression, or not depressed). Potentially confounding factors were controlled for in all three models. These included father's age, father's highest academic qualification (no qualification/GCSE/A-levels; diploma/equivalent; degree; and postgraduate degree), ethnicity (white and non-white), employment status (full-time; part-time; and unemployed/student), whether the father had other children, child's gender (male and female), child's birth weight and a diagnosis of postnatal depression in the child's mother. In all three GLMs, the gamma distribution and the log link function were chosen over the gaussian, poisson and inverse gaussian distributional families, and the identity link function, based on Akaike information criterion (AIC) and Park test statistics (Manning and Mullahy, 2001). The results obtained were converted back to the cost scale to obtain marginal effects.

## 3 Results

Of 4106 fathers who consented to participate in the study, a total of 1562 (38.04%) responded to the EPDS screening questionnaire at 7 weeks postpartum, of whom 1335 (85.6%) scored < 10 and 199 (12.74%) scored ≥ 10. A total of 28 (1.79%) fathers were excluded due to completely missing items on the EPDS questionnaire. A random selection of the EPDS low scorers (n = 266) and all the EPDS high scorers (n = 199) were contacted to obtain permission to be assessed using the SCID diagnostic tool for depression. A total of 273 (59%) fathers (n = 125 and n = 148 of the EPDS high and low scorers respectively) were not assessed. Of these, 85 fathers could not be contacted despite repeated attempts, 147 fathers declined without giving any reasons and 41 fathers were excluded because their child was older than the required age.

A total of 192 fathers (approximately 5% of those who initially consented to participate and 12% of those who responded to the screening questionnaire) consisting of 74 EPDS high scorers and 118 EPDS low scorers were assessed using the SCID diagnostic tool. Thirty-one (16%) fathers met the DSM-IV criteria for depression while 94 (49%) fathers failed to meet these criteria. Sixty-seven (35%) fathers were not currently depressed, but were deemed at high risk of developing depression.

In comparison to fathers in the high risk group and to the fathers without depression, fathers with depression incurred higher costs due to GP contacts, psychologist contacts, day care hospital (mental health) attendances and hospital outpatient attendances (Table 1). In comparison to fathers without depression, fathers in the high risk group incurred higher cost due to GP and psychologist contacts, physical day care hospital attendances and medical/surgical ward admissions. Overall, fathers with depression in the postnatal period incurred higher total father–child dyad costs (£1103.51) than fathers at high risk of developing postnatal depression (£1075.06), who in turn incurred higher total father–child dyad costs than fathers without depression (£945.03). A statistically significant cost difference in community care services (mean cost difference = £95.37; P = 0.034; Table 1) was observed between fathers with and without depression. Furthermore, Table 2 shows that after controlling for potentially confounding factors, fathers with depression incurred significantly higher community care service costs than fathers without postnatal depression (mean cost difference = £131.88; P = 0.005). Similarly, fathers at high risk of developing depression incurred significantly higher community care service costs in comparison to fathers without depression (mean cost difference = £81.95; P = 0.024; Table 2).

## 4 Discussion

Paternal depression in the postnatal period is not a widely recognised phenomenon and very few studies have attempted to study this problem. However there is growing evidence that men can suffer depression following the birth of a child and, like other mental illnesses, this is likely to have economic consequences; hence the need for investigations to improve understanding of the economic implications of the disease.

The main finding of this study suggests that depression in fathers in the postnatal period is associated with increased healthcare costs. In terms of incremental costs for each category of care, depression was associated with significantly higher community care costs. This finding is consistent with an earlier study on the economic impact of maternal postnatal depression, which reported a statistically significant cost difference for community care services alone (Petrou et al., 2002a). Within this category, costs associated with increased contacts with general practitioners and psychologists made the highest contribution to the observed cost difference between fathers with and without depression.

### 4.1 Study limitations

In this study, costs were estimated from a healthcare system perspective. We recognise that this may represent too narrow a perspective as the condition may impact upon other sectors of the economy, as well as upon the individuals themselves and their carers. However as one of the aims of this study was to stimulate further research into cost-effective preventative measures, we have adopted the perspective recommended by NICE for health technology appraisal purposes (NICE, 2008).

The costs reported in this analysis should be interpreted as conservative estimates for the following reasons. First, the cost calculations were based on self-reported consumption of healthcare services. Items of resource-use were collected over a period of 12 months and some degree of recall bias may have occurred, particularly for community care services in which a recall time frame of 6 months or less is generally considered optimal (Bhandari and Wagner 2006). While this is a limitation of the resource-use data resulting in possible underestimation of cost estimates, we have no reason to believe that underestimation of resource-use and costs varied across the three study groups (Petrou et al., 2002b; Van Den Brink et al., 2004). Nevertheless, given this limitation, caution should be exercised in extracting data from this study for use in secondary analyses.

Second, approximately 17% of total cost data were missing due to the failure of many fathers to complete relevant items of the resource-use questionnaire. Multiple imputation methods were used to generate cost estimates for the missing resource components. Although the fathers with missing information did not differ significantly from those with complete data in terms of socio-demographic characteristics and depressive status, we may not have adequately accounted for the potentially greater degree of uncertainty surrounding economic estimates had the imputed values actually been observed.

Finally, due to the relatively low response rates during the recruitment of participants, the study sample was of a smaller size than ideally required for an economic analysis (Strum et al. 1999), and may in part explain the failure to detect statistically significant differences in some cost estimates between the study groups.

### 4.2 Conclusion

The finding of this study provides preliminary insights into the economic impact of paternal depression in the postnatal period. We demonstrate that paternal depression is associated with higher community care costs. With a growing number of studies demonstrating the clinical impact of paternal depression in the postnatal period, this study highlights the need for further research into the economic impact of the disease in order to aid cost-effective allocation of resources for the treatment and prevention of paternal depression in the postnatal period.

## Role of funding source

This project is supported by a Wellcome Trust clinical research fellowship to PGR (078434).

## Conflict of interest

IPE is currently a PhD student at the Centre for Health Economics, University of York. This study was undertaken in partial fulfilment of the MSc degree at City University, London.

## Figures and Tables

**Table 1:** Mean cost incurred by the three study groups (£ sterling, 2008 prices).

	All three study groups				Comparison between fathers with and without PND		
Health care services	PND fathers (n = 31) Mean (s.d.)	High risk fathers (n = 67) Mean (s.d.)	Fathers without PND (n = 94) Mean (s.d.)	P-value^a^	Mean cost difference	95% CI^b^	P-value^c^
*Father*							
Community care services							
Midwife	27.07 (60.08)	22.21 (48.41)	22.41 (85.25)	0.942			
General practitioner	92.95 (176.02)	60.12 (110.37)	46.87 (82.13)	0.140			
Practise nurse	1.46 (2.63)	2.12 (5.36)	2.43 (6.61)	0.714			
Practise Counsellor	0.77 (1.56)	1.49 (5.43)	0.93 (4.37)	0.668			
Psychologist	23.47 (103.27)	14.24 (55.90)	8.21 (27.77)	0.412			
Physiotherapist	8.12 (18.48)	16.39 (43.38)	12.30 (54.84)	0.702			
Other service^d^	44.51 (87.85)	9.75 (28.01)	9.82 (32.49)	0.001			
Total community services	198.37 (246.48)	126.33(158.95)	103.00 (203.10)	0.067	95.37	12.66 to 205.49	0.034^e^
Day hospital							
Physical health	33.92 (71.93)	76.90 (201.51)	23.02 (64.77)	0.035			
Mental health	20.98 (93.25)	3.53 (9.68)	3.46 (11.07)	0.069			
Total day hospital	54.91 (115.58)	80.44 (203.07)	26.66 (70.95)	0.052	28.25	−8.99 to 76.52	0.107
Hospital outpatient							
Accident and emergency	15.66 (34.32)	15.36 (34.28)	20.89 (65.80)	0.771			
Other outpatient services^f^	57.74 (174.76)	26.48 (69.80)	12.27 (32.58)	0.034			
Total hosp. outpatient	73.40 (180.81)	41.84 (89.99)	33.16 (75.02)	0.176	40.23	−8.99 to 125.22	0.081
Hospital inpatient							
Medical/surgical ward	95.45 (244.90)	108.37 (234.88)	121.54 (592.59)	0.957			
Mother and baby unit	2.71 (6.59)	2.31 (6.90)	3.03 (15.61)	0.932			
Total hospital inpatient	98.16 (249.57)	110.68 (238.66)	124.56 (592.99)	0.955	−26.40	−192.73 to 109.76	0.810
Total (fathers only)	424.83 (560.45)	359.29 (517.97)	287.39 (686.53)	0.513	137.45	−94.91 to 380.87	0.315

*Child*							
Community paediatrician	124.21 (256.43)	220.94 (813.17)	139.03 (266.50)	0.557			
Hospital paediatrician	68.21 (140.86)	88.34 (238.54)	59.02 (137.37)	0.593			
Accident and emergency	89.83 (118.66)	42.71 (72.66)	53.11 (86.38)	0.047			
Paediatric ward	324.44 (721.82)	175.45 (455.34)	292.05 (1014.58)	0.589			
SCBU^g^	53.56 (155.12)	179.61 (641.01)	101.18 (329.12)	0.364			
Physiotherapist	11.80 (24.04)	6.50 (20.91)	10.68 (42.40)	0.673			
Other child care services	6.61(23.99)	2.21 (6.52)	2.56(14.55)	0.334			
Total child services	678.67(1079.75)	715.76 (1176.20)	657.64 (1265.19)	0.956	21.03	−427.98 to 474.76	0.934

Total father–child dyad	1103.51 (1430.57)	1075.06 (1386.70)	945.03(1478.95)	0.796	158.47	−367.41 to 797.36	0.603

a F-test statistics.

b 2000 bias-corrected bootstrap confidence intervals (CI); PND-postnatal depression.

c Student's t-test statistics.

d Chiropodist, dermatologist, health visitor, dentist, consultant (gastroenterology), surgeon (for vasectomy).

e P < 0.05.

f Ultrasound, fracture clinic, dentist, dermatologist, diabetic clinic, X-ray diagnostic imaging, and vasectomy.

g Special care baby unit.

**Table 2:** Generalised linear models (dependent variable = community care service costs).

Independent variable	Coefficient (std. error^a^)	Marginal effect (std. error^a^)	P-value
High risk fathers^b^	0.636(0.2484)	81.95(36.33)	0.024⁎⁎
PND fathers^b^	1.023(0.2999)	131.88(46.46)	0.005⁎⁎
Fathers' age	0.0265(0.020)	3.42(2.77)	0.218
Full time employment^c^	−0.102(0.385)	−13.20(49.42)	0.789
Diploma/equivalent^d^	−0.289(0.3666)	−37.26(48.47)	0.442
Degree^d^	0.168(0.3289)	21.70(42.52)	0.610
Postgraduate^d^	−0.637(0.3398)	−82.12(48.70)	0.092
White^e^	−1.244(0.4792)	−160.33(74.55)	0.032⁎⁎
Married/living together^f^	0.741(0.5349)	95.52(70.73)	0.177
Child's birth weight	−0.434(0.1855)	−56.00(27.53)	0.042⁎⁎
Other children	0.023(0.2772)	3.03(35.72)	0.932
Male child^g^	0.049(0.2381)	6.36(30.59)	0.835
PND mothers^h^	−0.042(0.2481)	−5.41(31.99)	0.866

⁎⁎ P < 0.05; base groups:

a Delta method standard error.

b Fathers without PND.

c Part-time employment/students/no employment..

d No qualification/GCSEs/A-levels.

e Non-white/Non-Europeans.

f Single.

g Female child.

h If mother was not diagnosed with PND.
